# Development and evaluation of a self care program on breastfeeding in Japan: A quasi-experimental study

**DOI:** 10.1186/1746-4358-5-9

**Published:** 2010-08-23

**Authors:** Masayo Awano, Keiko Shimada

**Affiliations:** 1 Department of Health Development Nursing, Graduate Course of Nursing Science Kanazawa University, 5-11-80 Kodatsuno, Kanazawa, 920-0942, Japan; 2 Division of Health Science, Graduate School of Medical Sciences, Kanazawa University, 5-11-80 Kodatsuno, Kanazawa, 920-0942, Japan

## Abstract

**Background:**

Although the importance of breastfeeding is well known in Japan, in recent years less than 50% of mothers were fully breastfeeding at one month after birth. The purpose of this study was to develop a self-care program for breastfeeding aimed at increasing mothers' breastfeeding confidence and to evaluate its effectiveness.

**Methods:**

A quasi-experimental pretest-posttest design was conducted in Japan. The intervention, a breastfeeding self-care program, was created to improve mothers' self-efficacy for breastfeeding. This Breastfeeding Self-Care Program included: information on the advantages and basics of breastfeeding, a breastfeeding checklist to evaluate breastfeeding by mothers and midwives, and a pamphlet and audiovisual materials on breastfeeding. Mothers received this program during their postpartum hospital stay.

A convenience sample of 117 primiparous women was recruited at two clinical sites from October 2007 to March 2008. The intervention group (n = 55), who gave birth in three odd-numbered months, received standard care and the Breastfeeding Self-Care Program while the control group (n = 62) gave birth in three even numbered months and received standard breastfeeding care.

To evaluate the effectiveness of the Breastfeeding Self-Care Program, breastfeeding self-efficacy and breastfeeding rate were measured early postpartum, before the intervention, and after the intervention at one month postpartum. The study used the Japanese version of The Breastfeeding Self-Efficacy Scale Short Form (BSES-SF) to measure self-efficacy.

**Results:**

The BSES-SF score of the intervention group rose significantly from 34.8 at early postpartum to 49.9 at one month after birth (p < 0.01). For the control group, the score rose from 39.5 at early postpartum to 46.5 at one month after birth (p = 0.03). The early postpartum fully breastfeeding rate was 90% for the intervention group and 89% for the control group. At one month postpartum, the fully breastfeeding rate declined significantly to 65% for the control group compared to 90% for the intervention group (p = 0.02).

**Conclusion:**

Results indicate that the Breastfeeding Self-Care Program increased mothers' self-efficacy for breastfeeding and had a positive effect on the continuation of breastfeeding.

**Trial Registration Number:**

UMIN000003517

## Background

It is highly desirable that an infant is fully breastfed for the first six months after birth, and that breastfeeding continues for as long as possible after six months [1]. Not only the commencement of breastfeeding itself, but also the duration of breastfeeding, is considered important for the healthy development of both mother and child [2].

In Japan, the importance of breastfeeding is well known. In 2007, the Ministry of Health, Labor and Welfare Japan reported that 96% of women prior to childbirth wanted to breastfeed, and after childbirth almost all women started breastfeeding [3]. However, according to the same survey, only 42.8% of mothers were fully breastfeeding at one month after birth. The mean hospitalization period after normal childbirth in Japan is usually five to seven days. This period is rather long and unique to Japan. Therefore it is hypothesised that if mothers were supported appropriately and effectively during hospitalization, the breastfeeding rate in Japan would be higher. Furthermore, it is thought that during this period of care and post childbirth guidance by midwives the breastfeeding period would more closely match mothers' earlier intentions. The present situation has raised questions about support measures and efficacy of care by health professionals.

There are various reasons why mothers in general who initially want to breastfeed their infants discontinue breastfeeding; the most common reasons being feelings of inadequate milk supply, reduced confidence in breastfeeding, and lack of volition [4-6]. The 2007 survey by The Ministry of Health, Labour and Welfare Japan, also reported almost every other woman (48%) felt they had an inadequate milk supply [3].

These "feelings of inadequate milk supply" mean that although the supply of breast milk is sufficient, the subjective feeling of the mother is that she is not producing enough milk. This is the most common reason why the mother stops breastfeeding shortly after starting, irrespective of whether she is from a developed or developing country [7,8]. One explanation is that a mother becomes uneasy during breastfeeding wondering if her supply of breast milk is sufficient to meet her infant's demands, or whether the mother is unable to determine the reason why the infant is crying [9]. Blyth et al. reported a positive correlation between breastfeeding self-efficacy and the duration of breastfeeding [10]. If the mother's breastfeeding self-efficacy is high, it is easier for her to continue breastfeeding. Researchers also reported that mothers successful at breastfeeding were more confident in child raising, and at the same time had a stronger feeling of self-efficacy and role fulfilment as a mother [11,12].

Breastfeeding educational programs for mothers are considered most effective for improving the start of breastfeeding and the breastfeeding period [13-15], but training programs and practical support methods to increase breastfeeding self-efficacy have not been sufficiently evaluated.

In this study, a Breastfeeding Self-Care Program (BSC-Program) was created for mothers after hospital discharge so they could confirm and evaluate the status of their breastfeeding. The purpose of the BSC-Program was to increase the mother's confidence and to facilitate a longer duration of breastfeeding. Accordingly, the aim of this study was to evaluate the effect of the BSC-Program through changes in self-efficacy regarding breastfeeding and the breastfeeding rate.

### Study framework

The study framework was based on Bandura's self-efficacy concept from social cognitive theory. Self-efficacy is defined as believing in one's own possibility of fulfilling a specific action and being confident about performing the task [16]. Bandura categorized preceding factors for general actions as: 'outcome expectancy', what sort of result can be expected, and 'efficacy expectancy', expectations about efficacy as to whether a person can actually complete an action [17]. Belief of this efficacy expectancy is labelled as 'self-efficacy'. It is to be expected that after childbirth a mother is overcome with relief that the delivery progressed safely. However, for several days after delivery, self-efficacy concerning breastfeeding may become unstable due to psychological and physical stress, such as concern about milk supply, and postpartum fatigue. Frequent breastfeeding can promote mother's self-efficacy; she can nurse without discomfort especially if she holds her infant and the infant properly attaches to the breast. Subsequently, the mother reaches a stage of 'outcome expectancy'; that breastfeeding was accomplished without nipple pain. Belief in self-efficacy is an important variable for predicting actual actions [16]; when the mother learns from repeated successful experiences, the desired result of long-term continuation of breastfeeding can be achieved.

We developed the BSC-Program so that mothers had a tool with which they could appropriately understand and evaluate their breastfeeding and therefore be able to take the necessary actions. If introduction of the BSC-Program into the actual process of mother's breastfeeding contributed to a positive evaluation of breastfeeding by the mother, then we could expect that the mother's self-efficacy would increase, facilitating long term continuation of breastfeeding.

## Methods

### Design

A quasi-experimental longitudinal design using pre-test-intervention-post-test with a non-synchronized non-equivalent control group was employed. The BSC-Program was the intervention. We conducted: 1) a baseline survey for all participants; 2) an intervention group receiving the BSC-Program plus standard education; the control group received only standard education, and 3) a one month follow-up survey for all participants to evaluate program effectiveness for group comparisons. Mothers giving birth in odd-numbered months entered the BSC-Program intervention group. Mothers giving birth in even-numbered months entered the control group. Outcome measures were: mothers' breastfeeding self-efficacy and breastfeeding rates.

### Setting

We recruited at two obstetric hospitals (A and B) in the Hokuriku area of Japan for women giving birth from October 2007 to March 2008. The two hospitals had approximately 300 to 500 births per year. Hospital A was a Baby-Friendly Hospital certified by UNICEF and the World Health Organization. Six midwives worked at this hospital; three were International Board Certified Lactation Consultants. Hospital B was not designated as a Baby-Friendly Hospital but strongly supported breastfeeding. This hospital had four midwives; one of them was an International Board Certified Lactation Consultant. Both hospitals had almost the same breastfeeding policy that followed the BFHI "Ten Steps to Successful Breastfeeding". Infant formula and glucose water are often given to neonates during hospitalization in Japan even if it is not medically necessary. However, both hospitals in this study provided infant formula supplementation only when medically necessary.

### Participants

During the study period, there were 324 births in the two hospitals. Inclusion criteria were: planning on breastfeeding; healthy during the pregnancy and postpartum period; delivered a healthy term singleton infant and the baby's birth weight was over 2500 g and after discharge, received enough appropriate nutrition and got help from their family with household chores. The exclusion criteria were: multipara; acute or chronic disease; taking regular medication; did not have a home DVD player and those who were not planning a one-month check up at the same hospital where they gave birth.

From the total births occurring, 156 (48%) mothers were primiparas and of those 135 met the remaining inclusion criteria; however 18 were excluded based on the exclusion criteria. The remaining 117 primiparas were invited to the study; all agreed to participate.

Midwives at each hospital recruited participants during the daytime Monday through Friday. They visited mothers' rooms during their hospitalization on the fourth day to recruit for this study. Fifty seven women were recruited from hospital A and 60 from hospital B. The 55 mothers giving birth in odd-numbered months (November 2007, January and March 2008) were assigned to the intervention group. The 62 mothers giving birth in even-numbered months (October, December 2007 and February 2008) were assigned to the control group.

After mothers gave informed consent, midwives asked participants to complete the baseline survey. All participants wanted to breastfeed and commenced breastfeeding. They received standard breastfeeding education with face-to-face demonstrations from midwives sometime after day four and before discharge. The standard education for mothers included information about the importance of breastfeeding, teaching about baby's feeding cues, positioning and latch-on techniques. The intervention group received the standard education and the BSC-Program. The control group received only the standard education. All mothers were able to access lactation consultants on demand during hospitalization.

### Sample

**Figure 1:**
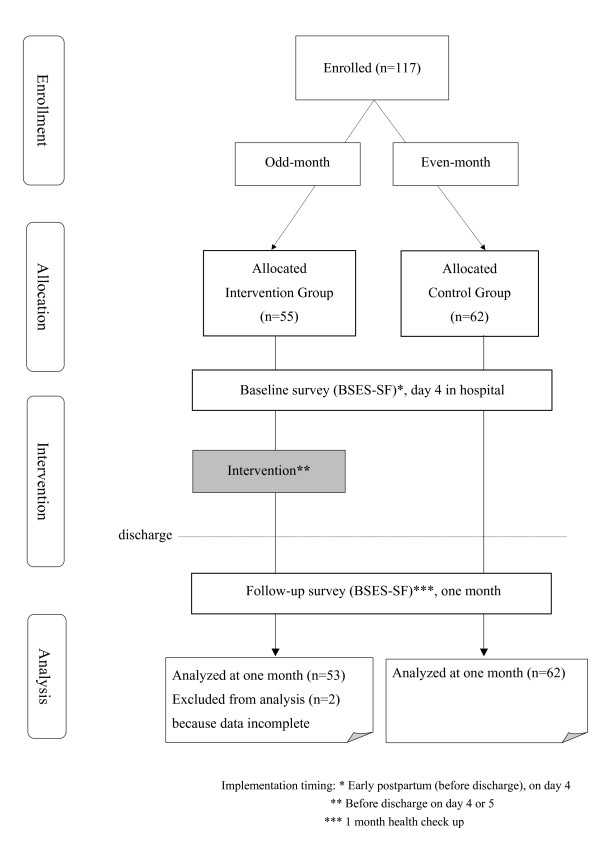
Participant flow chart

The participant pool varied based on the number of month deliveries in each hospital. Participation at baseline and follow-up was 100% (n = 117). However two participants who did not complete the questionnaire due to personal circumstances and were excluded from analysis, resulting in 53 participants in the intervention group and 62 participants in the control group (n = 115) (Figure 1).

A power analysis for a two-group test of mean differences to reach 80% power and a 0.80 effect size with an alpha value of 0.05 required a minimum sample size of 28 participants in each group [18]. Taking into consideration anticipated participant withdrawal; an estimated 40 participants for each group and the six months survey period were selected. The effectiveness of breastfeeding education program from a previous study was used as a reference to conduct the power calculation [19].

### BSC-Program

**Figure 2:**
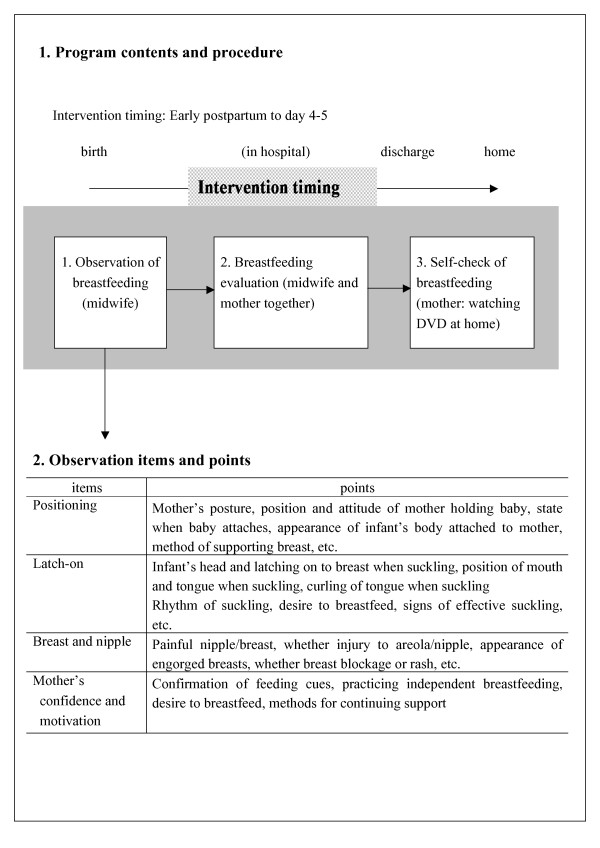
Outline and procedure for breastfeeding support program

The BSC-Program included a pamphlet and a DVD. The program focus was: advantages and basics of breastfeeding; baby's feeding cues; positioning; latch-on, and positive signs of baby's feeding (Figure 2). Mothers experienced with breastfeeding provided comments about the clarity and content of the BSC-Program.

The contents of the single page, double-sided breastfeeding pamphlet were based on the recommendations and supporting evidence from the UNICEF [20] and the American Academy of Pediatrics [2]. The pamphlet had a breastfeeding self-checklist that contained 14 points selected in accordance with observations from evidence-based publications [21-23]. The items included: mouth is wide open, chin is touching the breast, baby sustains a rhythmic 'suckle/swallow/breathe' pattern with periodic pause, swallowing is audible, and arm and hands are relaxed. The mother and midwife confirmed if the mother knew where and how to find on-going support. The content of the DVD was similar to the pamphlet. It provided an explanation of the advantages and basics of breastfeeding, baby's feeding cue, positioning, latch-on, positive signs of baby's feeding and the self-check list for breastfeeding. The DVD was a 10-minute PowerPoint slide show with audio explanation of the content. It was produced in a university audio laboratory with technical assistance.

The BSC-Program was given to mothers four or five days postpartum (after completion of base-line survey) prior to discharge. Mothers and midwives observed and assessed breastfeeding using the breastfeeding self-checklist in the pamphlet. Mothers also confirmed their breastfeeding knowledge and condition by completing the checklist in the pamphlet in interaction with the midwives.

Midwives encouraged the mothers to watch the DVD then evaluate their breastfeeding using the checklist at least once after discharge when they were able to review the materials at their leisure. They were instructed to visit the breastfeeding outpatients department in case they experienced concerns or difficulty about breastfeeding.

### Measures

Measures included three questionnaires. To evaluate mothers' breastfeeding confidence, the Breastfeeding Self-Efficacy Scale Short Form (BSES-SF) developed and psychometrically established by Dennis [24] was used. The BSES-SF includes psychological aspects of breastfeeding. It is a 14 item, five-point Likert scale ranging from 1 (*not at all confident*) to 5 (*very confident*), with a total score of 70. Total scores were used to calculate each respondent's self-efficacy. The scale's reliability was highly acceptable (Cronbach's alpha co-efficient of 0.90) [25]. Permission to use the BSES-SF Japanese version for this study was obtained from Dennis, and from Ohtsuka who had translated it into Japanese [26].

The second questionnaire, developed by the researchers, contained six sections; (a) while pregnant, what had been their intention about feeding their new baby, using a single item rated on a 3-point Likert scale: (1) will give either breast milk or formula; (2) will give breast milk if I can and (3) will give breast milk at any cost; (b) a visual analogue childbirth satisfaction scale; (c) amount of time for mother-child contact (skin-to-skin) immediately after birth; (d) amount of time for mother and child rooming-in; (e) water and nutritional supplementation other than breast milk (multiple-choice answer) and (f) frequency of accessing a lactation consultant.

The third survey was administered to the intervention group to evaluate the content and usefulness of the BSC-Program. It had 14 items, with scoring on a 4-point Likert scale: 1 (*poor or useless*) to 4 (*very good or very useful*). It also asked about the number of times participants watched the DVD, read the pamphlet and used the breastfeeding self-check checklist.

### Pilot survey

Three mothers participated in a pilot survey of the BSC-Program to confirm their understanding of the pamphlet, audiovisual materials and the questionnaire contents. This included checking that there were no impediments to the survey, such as difficulty using the audiovisual materials at home, ensuring that the image was clear and that the sound was easy to hear.

### Data collection

Participants were surveyed twice, once during early postpartum in hospital (baseline survey) and again at the health check-up one month after birth (follow-up survey). The baseline survey was administered and collected on day four after birth in the hospital. Midwives left survey questionnaires at the hospitals; hospital-based collaborators collected the completed questionnaires.

### Data analysis

Statistical analysis was conducted using SPSS 16.0 for Windows. Descriptive analysis (proportions) was used to characterize the overall samples. For the characteristics of participants, average ± standard deviation and frequencies (percentage) were used. For measuring breastfeeding self-efficacy, means ± standard errors were used and for distributions in the relationship between these two factors, Pearson's correlation coefficient (r), and Spearman's rank correlation coefficient (rs) were used. For comparing the intervention and control groups, a paired t-test, and two-way factorial ANOVA were used for parametric testing. For non-parametric testing the chi-square test was used to compare breastfeeding rate. A p value of 0.05 or less was considered significant.

### Operational definition of terminology

In this study, "fully breastfeeding rate" is defined as no formula was given; many infants were given glucose water after birth.

### Ethical standards

The Kanazawa University Medical Ethics Committee approved this study. All participants were informed of protection of their rights as research participants, including assurances of confidentiality and their right to withdraw from the study at any time without jeopardizing their care.

## Results

The response rate was 100% (n = 117). We excluded two mothers with incomplete responses; 115 responses (effective response rate 98%) were analyzed.

### Characteristics of participants

No difference was found between the intervention and the control group in terms of age, delivery type, average gestation period and infant birth weight (Table 1). All mothers commenced breastfeeding immediately after birth. Sixty mothers (57%) had contact with the lactation consultant after discharge. The mean number of contacts was 0.53 (range 1-6); there was no difference between two groups.

**Table 1:** Characteristics of participants and main outcome measures in comparison intervention and control groups

	Intervention group (n = 52)		Control group (n = 63)	
Mother's age (year) Delivery type vaginal caesarean forceps delivery	30.3 ± 0.6 (20-38)		28.9 ± 0.4 (20-36)	
	40 (77%)		48 (77%)	
	5 (10%)		8 (12%)	
	7 (13%)		7 (11%)	

Infant gestation weeks	39.3 ± 0.2 (36-42)		39.2 ± 0.1 (36-41)	

Infant birth weight (g)	3059.3 ± 60.1 (2340-3990)		3037.1 ± 50.9 (2340-3896)	

	day 4 postpartum	1-month postpartum	day 4 postpartum	1-month postpartum

BSES-SF score	34.8 ± 1.1 (24-48)	49.9 ± 0.9 (38-68)	39.5 ± 1.5 (20-55)	46.5 ± 1.7 (27-65)

Changes BSES-SF score day 4 to 1-month	15.1 ± 1.03 (1-30)		7.0 ± 1.26 (13-19)	

Fully breastfeeding n (%)	46 (90%)	46 (90%)	56 (89%)	41 (65%)

Age, delivery type, infant gestation weeks and infant birth weight are average ± standard deviation.

BSES-SF score is mean ± standard error.

### Comparison of breastfeeding self-efficacy

**Figure 3:**
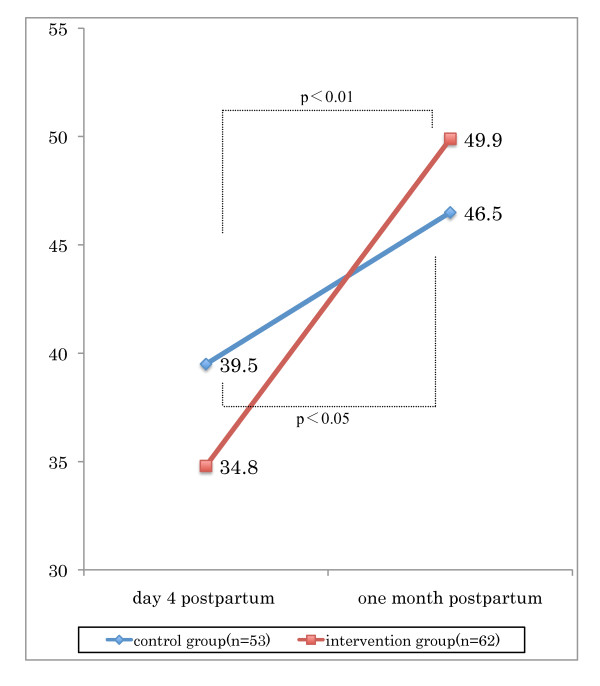
**Changes in breastfeeding self-efficacy and effect of program implementation from early postpartum to one month after birth**. Values are mean. Comparing intervention and control group on day 4 as early-postpartum and one month postpartum performed by paired t-test.

The mean BSES-SF score in the intervention group at baseline was 34.8 (SE = 1.1, range 24 to 48) and the control group was 39.5 (SE = 1.5, range 20 to 55) (Table 1). At baseline, the BSES-SF score in the control group was higher than the intervention group (ANOVA, F = 5.48, p < 0.05). The BSES-SF score in the intervention group, from baseline to follow-up increased significantly 34.8 (SE = 1.1, range 24-48) to 49.9 (SE = 0.9, range 38-68) (ANOVA, F = 5.48, p < 0.01). The control group's baseline to follow-up was 39.5 (SE = 1.5, range 20-55) to 46.5 (SE = 1.7, range 27-65) (ANOVA, F = 10.71, p < 0.05). The BSES-SF score was higher in the intervention group compared to the control group at one month follow-up. Furthermore, the changes in the BSES-SF score from baseline to follow-up increased more significantly for the intervention group (15.1, SE = 1.03, range 1 to 30) than for the control group (7.0, SE = 1.26, range -13 to 19) (ANOVA, F = 18.46, p < 0.01). Figure 3 shows the relationship between the groups and the timing (Figure 3). There was no difference between the two groups in the number of times lactation consultants were accessed after discharge.

The BSES-SF score for all participants (n = 115) at the baseline survey was 36.9 (SE = 1.0, range 20 to 55), and at the follow-up survey was 48.0 (SE = 1.1, range 25 to 68) increasing significantly by 10.5 (SE = 1.0, t = 10.7, p < 0.01). Of the 115 mothers, six had lower scores at one month than at baseline, all of whom were in the control group; none of the six had contacted the lactation consultants or received advice from the midwives.

### Analyses of breastfeeding rate

There was no difference between the two groups in the proportion of women fully breastfeeding at baseline: 90% (n = 46) in the intervention group and 89% (n = 56) in the control group. The fully breastfeeding rate for the intervention group was the same at follow-up as at baseline: 90% (n = 46). While the proportion fully breastfeeding in the control group decreased from 89% baseline to 64.8% (n = 41) on follow-up (chi-square test, p < 0.05). No mothers used only infant formula at baseline or follow-up. In both groups, contact with a lactation consultant was not associated with fully breastfeeding at follow-up.

### Perceived usefulness of BSC-program by the intervention group

Of the 55 mothers in the intervention group, 44 (80%) returned the evaluation about the content and usefulness of the BSC-Program. The mean number of times watching the DVD was 1.4 (range 1-3); the mean number of times reading the pamphlet was 2.5 (range 1-4) and the mean number of times using the breastfeeding self-check checklist was 2.0 (range 1-4).

The majority of women in the intervention group found the BSC-Program very helpful. The following responses were from mothers who answered 4 (*very good or very useful*) and 3 (*good or useful*). All 44 (100%) participants evaluated the BSC-Program stated: "The audiovisual materials increased my knowledge of breastfeeding, and were useful for my understanding"; 41 (93%) "The materials helped me to continue breastfeeding"; 40 mothers (91%) responded, "The material helped enhancing my confidence about breastfeeding"; 40 (91%) "The materials were useful when actually doing breastfeeding"; 38 (86%) "The materials helped me to learn breastfeeding techniques" and 37 (67%) "The materials helped to reduce my concern about my milk supply".

## Discussion

### Self-efficacy

Women in the intervention group had a significantly greater increase in self-efficacy than women in the control group. The effects of the BSC-Program are in accordance with factors of Bandura's social awareness theory [17].

For example, the BSC-Program reminded the mothers of the merits of breastfeeding shortly after childbirth, helped maintain volition to breastfeed (maintenance of motivation), and due to the mothers' understanding about the finer points of direct breastfeeding the mothers actually had the experience of breastfeeding (achievement of the pursued action). Other rationale for positive effects of the BSC-Program include the mother could evaluate her own breastfeeding status; the mother was aware of whether or not she could perform the action (thought-action conversion), and there was continued encouragement and support from the midwife (verbal persuasion).

In UNICEF's 20-hour course titled Baby-Friendly Hospital Initiative [20], the content states that before a mother leaves a maternity facility, she needs to be able to: feed her baby, understand the importance of exclusive breastfeeding for 6 months and continued breastfeeding after the introduction of complementary foods for two years and beyond, be able to recognize that feeding is going well and find out how to get the on-going support that she needs. The BSC-Program requirements in this study contain the same criteria. The American Academy of Pediatrics states that the breastfeeding period is extended when the mother receives appropriate intervention and continuing support and evaluation [2].

A number of studies have examined the effects of breastfeeding education, finding various methods to be effective [13,27-29]. These studies collectively show that a training program run by specialists is the most effective method to improve the commencement of breastfeeding and its duration. The BSC-Program developed for this study increased the breastfeeding self-efficacy of mothers, indicating that the contents of this Program provided effective education materials for the mothers.

Six mothers for whom the BSES-SF score declined, from the time of birth to one month later, had only experienced the standard education and none had accessed the lactation consultant. Only two of those mothers started giving infant formula as well as breastfeeding during the first month. While it was outside the scope of the survey to query mothers about why they lost confidence or why they started giving formula, previous researchers have addressed this issue. Factors reported as making it difficult to continue breastfeeding included: feelings of an inadequate milk supply, infant had suckling difficulty, nipple soreness and injury, and a lack of ongoing support [8,30-32]. It could be surmised that those mothers who had lower confidence at one month postpartum might have had some of the above problems with breastfeeding.

### Breastfeeding rate

The intervention group fully breastfeeding rate at one-month was significantly higher than the control group. This indicated that the BSC-Program was an effective and practical support method to increase breastfeeding rates, and could be considered clinically effective.

In this study, 72.2% mothers continued full-breastfeeding for one month, which was a much higher rate than the 2007 42.8% rate [3]. This may be due to our selection of hospitals that provided strong support breastfeeding for survey participants.

### Content of BSC-program

The majority of respondents evaluated the BSC-Program content positively and as generally acceptable as a breastfeeding self-care program. This appears to be the first self-care program created in Japan that evaluated breastfeeding continuation from the viewpoint of breastfeeding self-efficacy; thus it is hoped the study results may make a valuable contribution to breastfeeding care.

### Implication for practice

A crucial aspect of this program was the mutual and interactive observation and assessment of breastfeeding by mothers and midwives. By using the BSC-Program, practicing midwives reinforced their knowledge and skill about breastfeeding support. From the point of view of enabling joint use between midwives and mothers, this program should also be suitable for clinical application and for easy implementation over a short period. However, for practical purposes, the BSC-Program needs to be refined for easier use such as making it accessible through a web site for mobile telephones or computer, as well as a non-technology based version. Also this program needs periodic updating based on user feedback.

### Limitations

This study was a quasi-experimental intervention research lacking random selection. The hospital population may not have reflected the general population of women giving birth. Women with no access to DVD playback systems were excluded. Data were based on the mothers' subjective views. Consequently, the results were affected by the personality and environment of the mother, the progress of the pregnancy and delivery, and support conditions after hospital discharge. Larger random controlled trials are needed to control for threats to internal and external validity so that results can be generalized. Qualitative studies are needed to gain a deeper understanding of those mothers who did not continue fully breastfeeding after one month.

These survey results are limited to certain hospitals and participants, and the midwives who implemented this program were lactation consultants. It is possible that the experience of midwives capable of appropriate observation and evaluation of breastfeeding affected the results of this program. Furthermore, the study period should be extended beyond one month after birth to evaluate the effects of long-term continuation of breastfeeding.

## Conclusions

We developed a breastfeeding self-care program with the aim of increasing breastfeeding self-efficacy after childbirth, and to facilitate continuation of breastfeeding. The BSC-Program comprised evaluation of breastfeeding by the mother and the midwife, and a breastfeeding pamphlet and audiovisual materials for the mother. The effects of implementation of the BSC-Program made it clear that the evaluation indices comprising the mother's self-efficacy and the breastfeeding rate were both significantly higher for the BSC-Program implementation group compared to the control group.

## Abbreviations

BSES-SF: The Breastfeeding Self-Efficacy Scale Short Form; BSC-Program: Breastfeeding Self-Care Program

## Competing interests

The authors declare that they have no competing interests.

## Authors' contributions

MA carried out the study and wrote the manuscript. KS supervised MA in her pre-doctoral work and in her post-doctoral fellowship and worked closely with her preparing the manuscript. Both authors read and approved the final manuscript.
